# Regionally specific levels and patterns of keratin 8 expression in the mouse embryo visceral endoderm emerge upon anterior-posterior axis determination

**DOI:** 10.3389/fcell.2022.1037041

**Published:** 2022-12-01

**Authors:** Evangéline Despin-Guitard, Ronan Quenec’Hdu, Wallis Nahaboo, Nicole Schwarz, Rudolf E. Leube, Claire Chazaud, Isabelle Migeotte

**Affiliations:** ^1^ Institut de Recherche Interdisciplinaire en Biologie Humaine et Moléculaire (IRIBHM), Université Libre de Bruxelles, Brussels, Belgium; ^2^ Université Clermont Auvergne, CNRS, INSERM, GReD Institute, Faculté de Médecine, Clermont- Ferrand, France; ^3^ Institute of Molecular and Cellular Anatomy, RWTH Aachen University, Aachen, Germany

**Keywords:** mouse embryo development, extra-embryonic tissues, keratin intermediate filaments, epithelia, cytoskeleton

## Abstract

The mechanical properties of the different germ layers of the early mammalian embryo are likely to be critical for morphogenesis. Cytoskeleton components (actin and myosin, microtubules, intermediate filaments) are major determinants of epithelial plasticity and resilience to stress. Here, we take advantage of a mouse reporter for Keratin 8 to record the pattern of the keratin intermediate filaments network in the first epithelia of the developing mouse embryo. At the blastocyst stage, Keratin 8 is strongly expressed in the trophectoderm, and undetectable in the inner cell mass and its derivatives, the epiblast and primitive endoderm. Visceral endoderm cells that differentiate from the primitive endoderm at the egg cylinder stage display apical Keratin 8 filaments. Upon migration of the Anterior Visceral Endoderm and determination of the anterior-posterior axis, Keratin 8 becomes regionally distributed, with a stronger expression in embryonic, compared to extra-embryonic, visceral endoderm. This pattern emerges concomitantly to a modification of the distribution of Filamentous (F)-actin, from a cortical ring to a dense apical shroud, in extra-embryonic visceral endoderm only. Those regional characteristics are maintained across gastrulation. Interestingly, for each stage and region of the embryo, adjacent germ layers display contrasted levels of keratin filaments, which may play a role in their adaptation to growth and morphological changes.

## Introduction

During early mouse embryogenesis, the first lineage separation events also give rise to the first epithelial barriers (Thowfeequ et al., 2022). At the eight-cell stage (embryonic day (E) 2), blastomeres already display some epithelial features, such as polarity illustrated by an apical domain rich in microvilli (Ducibella et al., 1977), cortical tension, and E-cadherin accumulation at cell-cell interfaces. As embryos undergo compaction, cells acquire distinctive features depending on their exposed surface area, notably differential YAP localization (Royer et al., 2020). During subsequent divisions, outer cells differentiate as extra-embryonic trophectoderm (TE), the first fully polarized epithelium, while nonpolar and highly contractile cells remaining inside form the inner cell mass (ICM) (Chazaud and Yamanaka, 2016). Sealing of junctions between TE cells allows blastocyst cavity expansion; the TE then becomes subdivided into polar TE covering the ICM and mural TE overlaying the cavity at the abembryonic pole (Christodoulou et al., 2019). At the late blastocyst stage (E4), the ICM segregates into two tissues that subsequently epithelialize: the epiblast, source of most embryonic tissues, and the primitive endoderm, precursor for parietal and visceral endoderm (VE). Implantation occurs around E5. Upon amniotic cavitation of the epiblast and elongation of the embryo, the VE covers both the proximal trophectoderm-derived extra-embryonic ectoderm and the distal epiblast. At the extremity of the cylinder, a subset of VE cells change shape from squamous to columnar and form an organizer known as the distal visceral endoderm (DVE) (Stower & Srinivas, 2014). The DVE then becomes the motile anterior visceral endoderm (AVE), which undergoes collective migration towards the embryonic/extra-embryonic boundary around E5.5 and thereby defines the anterior-posterior axis of the embryo. Signaling from the AVE restricts a permissive region for gastrulation on the posterior side of the E6.5 embryo. At the primitive streak (PS), cells delaminate from the epiblast to become mesoderm and endoderm (Ferretti and Hadjantonakis, 2019; Nowotschin et al., 2019). Mesoderm cells originate from epiblast cells all along the PS, undertake a complete epithelial-mesenchymal transition (EMT), and actively migrate either proximally as extra-embryonic mesoderm, or laterally as mesodermal wings in the embryonic region. Definitive endoderm cells arise in the distal PS through partial EMT (Probst et al., 2020), move within the mesodermal wings *via* a still uncharacterized mechanism, then go through mesenchymal-epithelial transition to intercalate between VE cells (Ferrer-Vaquer et al., 2010).

The three cytoskeletal components: microtubules, actomyosin, and intermediate filaments, display extensive remodeling and crosstalk during early mouse embryogenesis (Lim and Plachta, 2021). Microtubules and actin filaments are present in all cells from the fertilization onwards; they become polarized upon compaction and accumulate in the apical region of the blastomeres. Among intermediate filaments, keratins (Krt or K) are the first to be expressed during mouse development. Transcripts for *Krt 7*, *8, 18,* and *19* were first detected at E1.5 day (Lu et al., 2005). Type II K7 and 8 proteins were identified by immunostaining as diffusely distributed aggregates throughout cells at the 8-cell stage (E2.5) (Lu et al., 2005; Ralston and Rossant, 2008). Between the 8 and 16-cell stages, apical keratin-based filament-like structures become detectable, likely because the expression of type I keratins K18 and 19 allows the formation of type I/II heterodimers. It was recently suggested that expression of K8 and 18 displays cell-to-cell variability prior to the segregation of inner and outer cells (Lim et al., 2020), which may play a role in fate determination. The dense actin network that promotes keratin apical localization in interphase may hinder redistribution of keratin filaments upon division, leading to asymmetric distribution between daughter cells (Lim et al., 2020). At the blastocyst stage, keratin filaments form an intricate network in the TE, while they are undetectable in the ICM. Post implantation, they are present in all extra-embryonic cells, independent of their germ layer of origin (Saykali et al., 2019).

The creation of a K8 reporter mouse line allowed documenting keratin network formation *in vivo* (Schwarz et al., 2015). A YFP-encoding sequence was inserted in frame at the end of the *Krt8* gene, thereby encoding a fusion Krt8-eYFP protein whose expression is under the native *Krt8* regulatory sequences. Live imaging of pre-implantation mouse embryos first detected a diffuse eYFP signal 6 h–8 h after compaction. Fluorescent dots (that were shown by immunofluorescence to co-localize with desmosome markers) accumulated at the cell borders. Over time, K8 positive dots became connected by filamentous elements, primarily at the membrane of outer cells at the morula stage, then in the cytoplasm at the blastocyst stage, progressively creating a dense web. The generation of a K8-containing mesh was also observed in extra-embryonic mesoderm cells surrounding the extra-embryonic cavity in late gastrulation embryos (Nahaboo et al., 2022).

Here, we recorded K8 expression pattern in the mouse embryo between the late blastocyst and the gastrulation stages. As expected, K8 was strongly present in the extra-embryonic ectoderm, and undetectable in the epiblast. K8 level and subcellular localization in the VE were highly dynamic; region-specific distributions for the keratin and actomyosin cytoskeletons emerged at the same place and time, with opposite changes in density.

## Materials and methods

### Mouse breeding and genotyping

Mouse colonies were maintained in a certified animal facility in accordance with European guidelines. Experiments were approved by the local ethics committee (“Commission d’éthique et du bien-être animal”) under protocol 725N. Mouse lines were CD1 wild-type (Janvier Labs) and K8-eYFP (Schwarz et al., 2015) bred on a CD1 background. Mouse genomic DNA was isolated from ear biopsies treated for 1 h at 95°C in NaOH 0.05M to simultaneously genotype and identify animals.

### Antibodies

The following primary antibodies were used: anti-Krt8 (rat, 1:100, TROMA-I-S, DSHB, AB_531826), anti-Vimentin (rabbit, 1:200, abcam ab92547), anti-Sox17 (Goat 1:100, R&D systems AF 1924), anti-TFAP2C (rabbit, 1:50, Santa Cruz, sc-8977), anti-Cer1 (goat, 1:500, R&D systems AF 1986). Rhodamine Phalloidin (1:500, abcam ab235138) was used to mark F-actin filaments, and nuclei were stained with DAPI (1:500, Sigma, D9542).

### Embryo recovery, staging, and pharmacological treatment

Embryos were recovered at the appropriate time point after observation of a vaginal plug at day 0. E3.5 and E4 embryos were flushed from the uterus, recovered in M2 medium (Sigma, M7167), and cultured in M16 medium (Sigma, M7292). E4.5 to E5.0 embryos were dissected out of the decidua in PBS using #5 forceps under a transmitted light stereomicroscope. From E5.5, embryos were dissected in Dulbecco’s modified Eagle medium (DMEM) F-12 (Gibco) supplemented with 10% Fetal Bovine Serum (FBS), 1% Penicillin/Streptomycin (P/S) and L-glutamine, and 15 mM HEPES, using #5 forceps and tungsten needles under a transmitted light stereomicroscope. Bright-field pictures of the litter or single embryo were taken before any manipulation to ensure adequate staging. For live imaging, embryos were cultured in 50% DMEM-F12 with L-glutamine without phenol red, 50% rat serum (Janvier), at 37°C and 5% CO_2_.

Embryo staging was based on the number of days after observation of a vaginal plug, morphological criteria, and markers when available. E4-E4.5 late blastocysts were undistinguishable by morphology only; they were classified as E4.5 upon completion of the separation between epiblast and primitive endoderm, visualized through immunostaining for Sox17. E5 embryos are characterized by the elongation of the epiblast, the formation of extra-embryonic ectoderm from polar TE, and the specification of VE and parietal endoderm from the primitive endoderm. At E5.5, embryos were classified according to the degree of migration of the AVE, which can be visualized either through morphology (AVE cells are cuboidal while the rest of the embryonic VE is squamous) or immunostaining for the AVE marker Cerberus 1 (Cer1). At E6.5 and 7.5, embryos were staged according to anatomical landmarks described in (Downs and Davies, 1993).

K8-eYFP embryos dissected in the morning of E6.5 were cultured in presence of Cytochalasin D reconstituted in ethanol and diluted to a final concentration of 5 μM in culture medium for 30 min prior to fixation and staining for F-actin and nuclei.

### Whole mount immunostaining

After dissection, embryos were fixed in 4% paraformaldehyde diluted in PBS for 1 h at 4°C. Immunostaining was performed in PBS containing 0.5% Triton X-100, 0.1% BSA, and 5% heat-inactivated horse serum. Embryos were then placed individually in a conical well (Ibidi 15 wells, art n° 8150). K8-eYFP primary fluorescence was preserved after fixation and therefore no antibody staining was required. Primary antibodies were incubated overnight at 4°C, and secondary antibodies were incubated for 2 h at room temperature. After secondary antibody incubation, embryos were washed in 1x PBS, then in 0.02 M phosphate buffer. E4-E5 immunostained embryos were treated with the clearing reagent scaleA2 (Hama et al., 2011). To enhance optical clarity, E6.5 and E7.5 embryos were left to dry for 1–2 min, then treated in 35 µl of Refraction Index Matching (RIM) solution (Treweek et al., 2015). RIM solution was prepared by dissolving 40 g of Histondenz (Sigma D2158) in 30 ml of 0.02 M phosphate buffer. To enable tissue adjustment, embryos were left in RIM over night at 4°C. Prior to image acquisition, RIM buffer was replaced by RI 1.46 silicone oil (Sigma #17633) to prevent the embryo from floating while maintaining refraction index matching. Embryos were imaged with a Zeiss LSM 780 microscope equipped with Plan-Apochromat 25×/0.8, C Achroplan 32×/0.85 and LD C Apochromat 40×/1.1 objectives, or Leica SP8 equipped with HC PL APO 40X NA:1.3 OIL CS2.

### Live imaging

After dissection, embryos were left to rest for 1 h at 37°C and 5% CO_2_ in culture medium. Embryos were then placed in conical wells (Ibidi) in culture medium and imaged under a Zeiss LSM 780 microscope equipped with a two-photon laser (Coherent) at 950 nm and Plan-Apochromat 25×/0.8, C Achroplan 32×/0.85 and LD C Apochromat 40×/1.1 objectives. Stacks were acquired every 15 min to every hour with a 3 or 5 µm Z-interval. Embryos were cultured for an additional 6 h–12 h after imaging to check for fitness.

### Image analysis

Images were analysed using Imaris, Arivis, and Fiji. To quantify the signals for K8-eYFP and F-actin (Phalloidin) in embryonic and extra-embryonic VE at E6.5, 10 µm-long lines were drawn at multiple locations of the VE and their intensity profiles were plotted using the Plot Profile function in Fiji. At least three different lines were plotted in the anterior and posterior embryonic and extra-embryonic regions of each embryo. We collected grey-values (arbitrary unit) in function of the distance. Distances from the posterior region were harmonized to obtain the same starting point (0) as the anterior region, and grey-values were averaged by distance. A ratio of data from the embryonic region to data from the extra-embryonic region was calculated.

## Results and discussion

**FIGURE 1:**
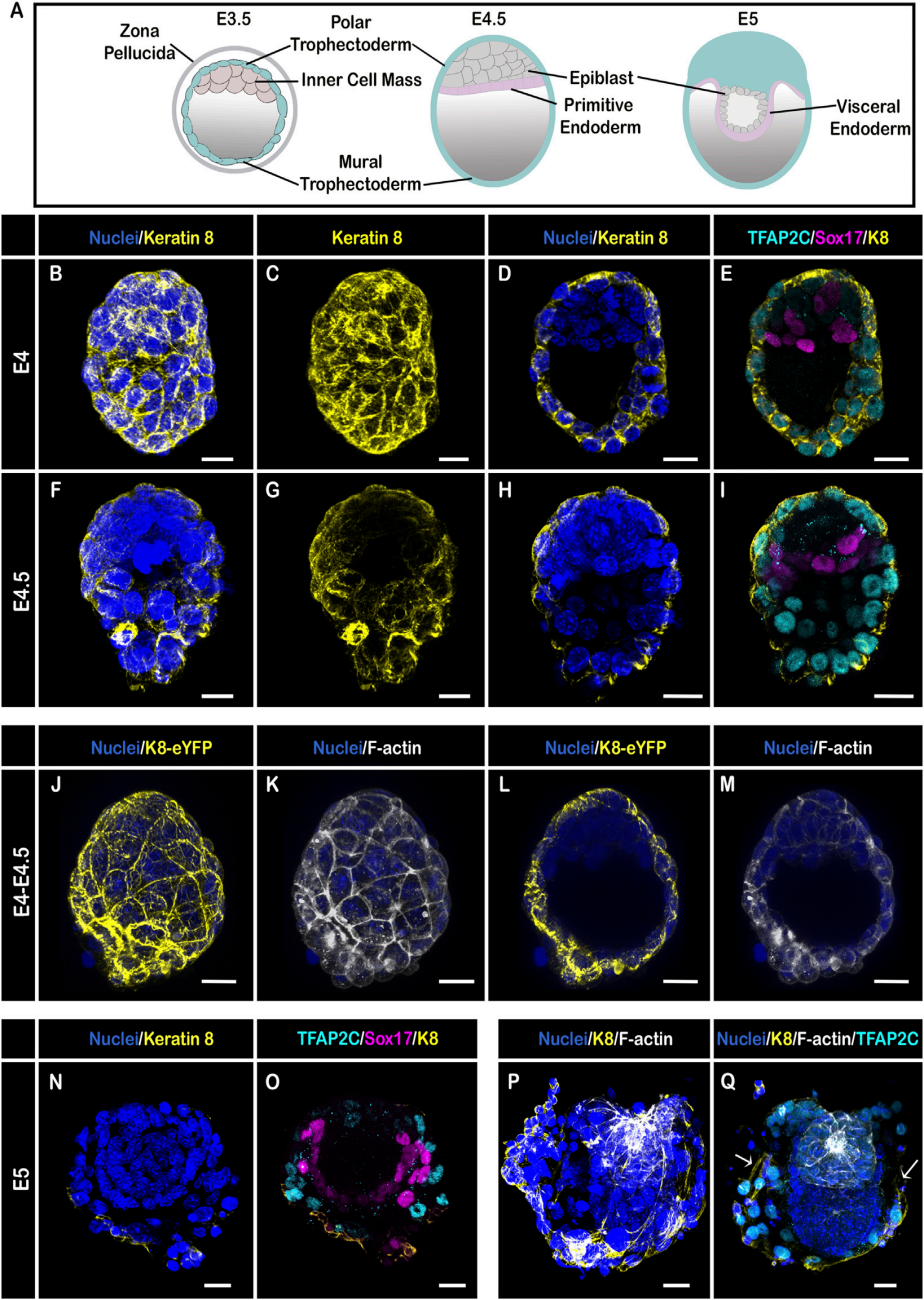
Keratin 8 organization in E4–E5 mouse embryos. **(A)** Schemes illustrating embryo stages and layers; **(B–I)** Confocal images acquired with a ×40 oil immersion objective from E4 **(B–E)** and E4.5 **(F–I)** embryos (*n* = 2 and 4, respectively) stained for nuclei (DAPI, dark blue), Keratin 8 (yellow), TE (TFAP2C, cyan) and Primitive Endoderm (Sox17, magenta). **(B,C,F,G)** are whole-mount 3D reconstructions, **(D,E,H,I)** are 3D projections from selected Z-slices. **(J–M)** Confocal images acquired with a ×40 water immersion objective from a E4-4.5 embryo bearing the K8-eYFP transgene (yellow) and stained for nuclei (DAPI, blue) and F-actin (Phalloidin, grey) (*n* = 6); **(J,K)** are whole-mount 3D reconstructions, **(L,M)** are 3D projections from selected Z-slices. **(N–Q)** Confocal images acquired with a ×40 oil immersion objective from 2 E5 embryos (*n* = 7) stained for nuclei (DAPI, dark blue), Keratin 8 (yellow), TE (TFAP2C, cyan) and Primitive Endoderm (Sox17, magenta); **(N,O,Q)** are 3D projections from selected Z-slices, **(P)** is a whole-mount 3D reconstruction. Arrows in **(Q)** show the limit of TE cells in contact with the endometrium. Scale bars = 20 µm.

K8 has been used as a TE marker for decades. At the late blastocyst stage, when the ICM has segregated into epiblast and primitive endoderm (Figure 1A), K8 was indeed strongly identified by immunostaining in TE cells (marked by TFAP2C), and remained undetectable in inner cells (Figures 1B–I; the primitive endoderm is marked by Sox17) (Sozen et al., 2019). Confocal microscopy of E4-4.5 embryos homozygous for the K8-eYFP knock-in transgene and stained for nuclei and F-actin confirmed the absence of expression of K8 in the ICM (Figures 1J–M; Supplementary Video S1). At E5 (Figures 1N–Q), when the embryo elongates within the blastocoel, K8 was still indistinguishable by immunostaining in early VE derived from primitive endoderm, and remained localised apically throughout the TE. Single cell RNA sequencing has uncovered an increased expression of keratins in mural, compared to polar, TE cells (Seong et al., 2022). We found that the level of K8 was higher, with a thicker region of expression apically and extended laterally, only in mural TE cells that are in contact with the endometrium (Figures 1P,Q).

**FIGURE 2:**
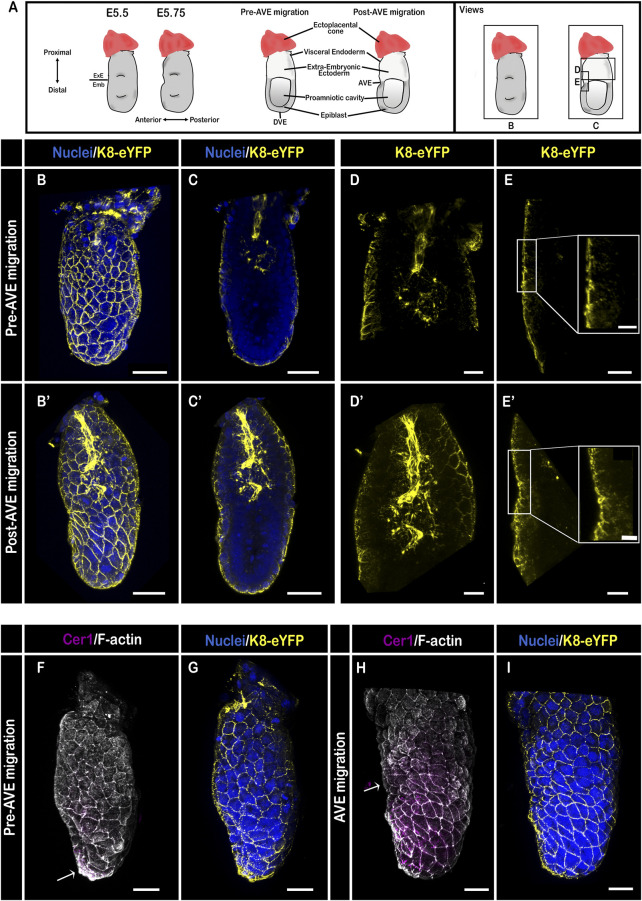
Keratin 8 organization in E5.5 mouse embryos. **(A)** Schemes of E5.5 embryo morphology, germ layers, orientation, and representation of angles of view. ExE: extra-embryonic, Emb: embryonic. **(B–E′)** Confocal images acquired with a ×40 water immersion objective from pre- **(B–E)**, and post- AVE migration **(B′–E′)** fixed embryos bearing the K8-eYFP transgene (yellow) and stained for nuclei (DAPI, blue) (*n* = 7). **(B,B′)** are whole-mount 3D reconstructions (scale bars = 50 µm), **(C,C′)** are 3D projections from selected Z-slices (scale bars = 50 µm), **(D,D′)** are 3D projection from selected Z-slices of the extra-embryonic region (scale bars = 20 µm), **(E,E′)** are 3D projections from selected Z-slices of the anterior side at the border between embryonic and extra-embryonic regions (scale bars = 20 µm; scale bars for zoom = 10 µm). **(F–I)** 3D reconstructions of confocal images acquired with a ×40 water immersion objective from pre- **(F,G)**, and AVE migration **(H,I)** embryos bearing the K8-eYFP transgene (yellow) and stained for nuclei (DAPI, blue), F-actin (Phalloidin, grey), and AVE (Cer1, magenta) (*n* = 4).

3D reconstruction of whole-mount K8-eYFP embryos at the egg cylinder stage (Figure 2A) prior to AVE migration (E5.25) revealed the appearance of a dense network of K8 containing filaments in the VE, with an increased density at cell-cell contacts [Figures 2B,G; the DVE is marked by Cer1 in Figure 2F (arrow)]. 3D reconstruction from selected Z-slices at the level of the cavity showed a strong YFP signal in the extra-embryonic ectoderm (Figure 2C), while the epiblast had no detectable K8 but started to express Vimentin (Supplementary Figures S1D). K8-eYFP appeared as large dots more abundant in the center of the ectoderm and thick bundles in the most proximal part continuous with the ectoplacental cone (Figure 2D). Focusing on the VE confirmed the homogenous pattern of expression all along the epithelium, with a higher level on the cells’ apical side, compared with the lateral side (Figure 2E; Supplementary Figures S1B,C).

Upon initiation of AVE migration (Figure 2H), the transgene allowed following cell shape changes in VE cells. It highlighted the contrast between the extra-embryonic VE, that retains a regular hexagonal arrangement, and the embryonic VE displaying a more diverse range of cell shapes as well as formation of rosettes (Figure 2I; Supplementary Videos S2, S3) (Migeotte et al., 2010; Trichas et al., 2012). There was no detectable fluorescence in the epiblast. The bundles in the center of the extra-embryonic ectoderm extended distally, concomitant with the expansion of the cavity (Christodoulou et al., 2018) (Supplementary Video S3). In VE cells that had changed shape from squamous to columnar, keratin filaments highlighted both the apical and lateral sides (Supplementary Video S3).

In embryos in which AVE migration was completed (Figure 2B′; Supplementary Video S4), a dense network of K8 filaments lined the cavity in the extra-embryonic region and extended in the ectoplacental cone (Figures 2C′,D′; Supplementary Video S4). A difference in the level and distribution of K8-eYFP emerged between VE cells on either side of the embryonic/extra-embryonic boundary (Figure 2E′), at which AVE migration stops (Figure 2A).

Among the molecular determinants of the abrupt barrier for AVE migration between the anterior epiblast and extra-embryonic ectoderm are distinct patterns of the Planar Cell Polarity (PCP) protein Disheveled (Dvl) 2 and the actomyosin cytoskeleton (Trichas et al., 2011). Indeed, prior to AVE migration, Filamentous (F)-actin levels are uniform across VE cells, where F-actin forms cortical rings [(Trichas et al., 2011), Figure 2F; Supplementary Figures S1A,D]. Upon AVE migration, specifically in the extra-embryonic VE, there is a reduction in Dvl2 expression while F-actin and Myosin IIA become enriched on the entire apical surface (Trichas et al., 2011, Figure 2H; Supplementary Figures S1E,H). Disruption of the PCP pathway perturbs Dvl2 and F-actin localization and causes AVE migration defects, notably an “overmigration” phenotype, in which cells are capable to cross the border (Trichas et al., 2011). Since we observed an opposite regulation of the keratin intermediate filaments network (Figures 2G,I) at the same stage and location, it would be interesting to record the expression pattern of K8 upon perturbation of the PCP and the actin cytoskeleton in E5.5 mouse embryos throughout AVE migration.

An inverse evolution of the density of the actomyosin and keratin cytoskeletons has been observed in other contexts. For example, epithelial cells submitted to extreme stretch display “superelasticity,” an unstable state linked to dilution of cortical actin that is rescued by a reinforcement of the keratin filaments network (Latorre et al., 2018). Conversely, in migrating cells, keratin and actin flows are linked, as the interplay between keratin and actin dynamics is modulated by the properties of the environment (Pora et al., 2020).

The difference in localization of F-actin was maintained in E6.5 and E7.5 embryos [(Trichas et al., 2011); Supplementary Figures S1E,H,I with quantification in K and L]. To record the pattern of K8-eYFP at E6.5 and E7.5, embryos were either cleared using Refraction Index Matching (RIM) buffer prior to whole-mount static confocal imaging or submitted to 2-photon live imaging (Supplementary Figure S2; Supplementary Videos S5, S6).

**FIGURE 3:**
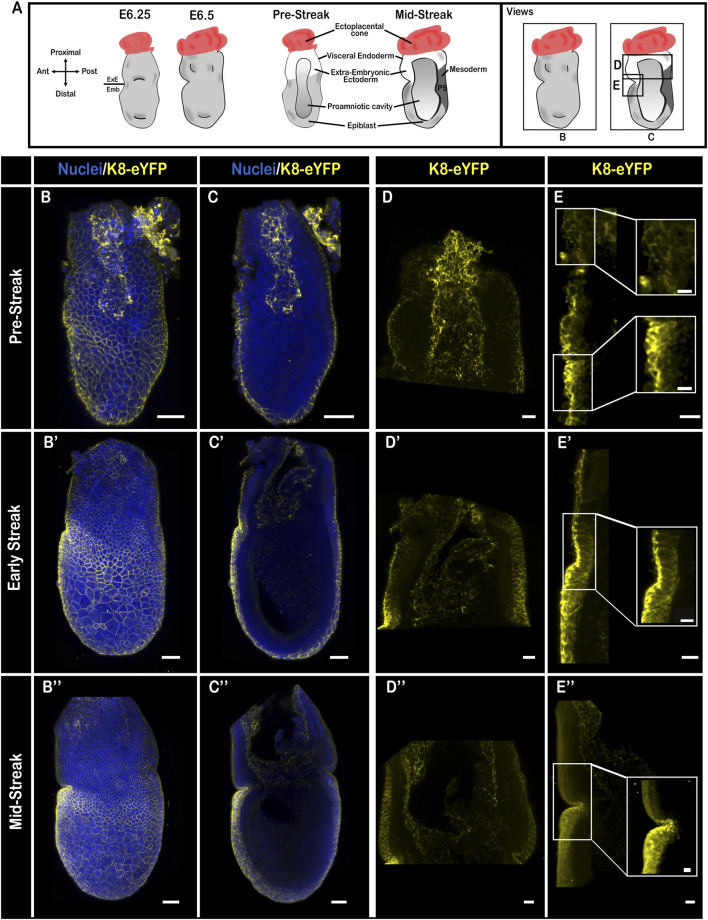
Keratin 8 organization in E6.5 mouse embryos. **(A)** Schemes of E6.5 embryo morphology, germ layers, orientation, and representation of angles of view. ExE, extra-embryonic; Emb, embryonic. **(B–E”)** Confocal images from Pre-Streak **(B–E)**, Early-Streak **(B′–E′)** and Mid-Streak **(B”–E”)** fixed embryos bearing the K8-eYFP transgene (yellow) and stained for nuclei (DAPI, blue). Embryos were cleared using the RIM buffer then mounted in conical wells (Ibidi) with 1.42 IR silicone oil and imaged by confocal microscopy with ×40 or ×25 water immersion objectives (*n* = 13). **(B,B′,B”)** whole-mount 3D reconstructions (scale bars = 50 µm); **(C,C′,C”)** 3D projections from selected Z-slices (scale bars = 50 µm); **(D,D′,D”)** 3D projection from selected Z-slices of the extra-embryonic region (scale bars = 20 µm); **(E,E,E”)** 3D projections from selected Z-slices of the anterior side at the border between embryonic and extra-embryonic regions (scale bars = 20 µm; scale bars for zooms = 10 µm).

In pre- and early-streak embryos (Figure 3A), K8 strongly marked cell-cell junctions in the embryonic VE, on the apical side of squamous cells, and on the apical and basal sides of columnar AVE cells. In contrast, it was more discrete and restricted apically in columnar extra-embryonic VE cells (Figures 3B–E′; Supplementary Figures S1F,G,J with quantification in K and L; Supplementary Video S7). This distinction was maintained upon perturbation of the actin cytoskeleton through treatment with cytochalasin D, a toxin that causes both disruption of existing filaments and inhibition of actin polymerization (Supplementary Figures S1M–P). The apical side of extra-embryonic ectoderm cells displayed strong labelling. In the epiblast, there were no detectable keratin filaments; expression of Vimentin was stronger than at E5.5 and enriched on the posterior side (Supplementary Figures S1G,H). At later stages of gastrulation (Figures 3B″–E″; Supplementary Figure S2B; Supplementary Videos S5, S8), the keratin network became denser, and more diffusely distributed (still with an apical reinforcement) in embryonic VE cells, particularly in the AVE cells close to the embryonic/extra-embryonic border in the prospective head region.

**FIGURE 4:**
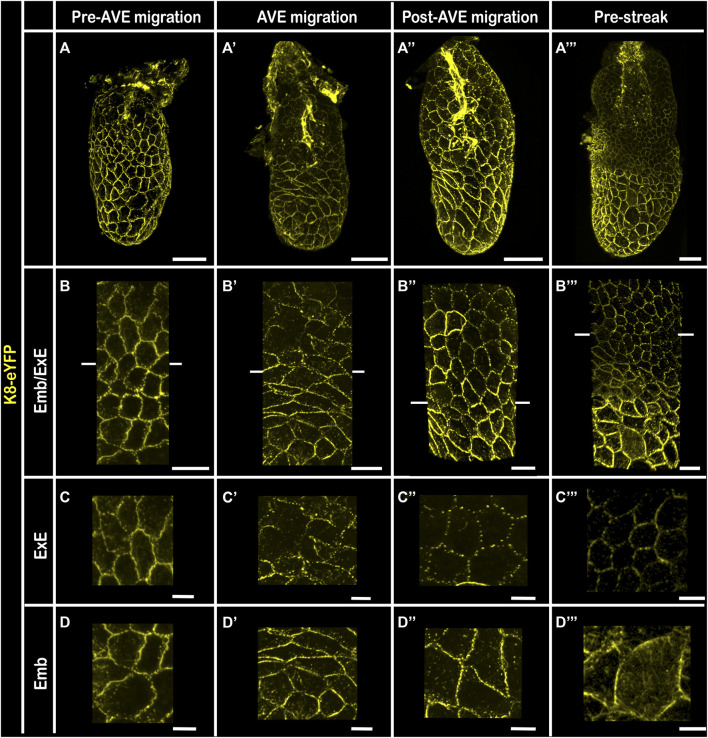
Keratin 8 expression pattern in embryonic versus extra-embryonic Visceral Endoderm 3D reconstructions of Pre-AVE migration **(A–D)**; AVE migration **(A′–D′)**; Post-AVE migration **(A”–D”)** and Pre-Streak **(A‴–D‴)** fixed embryos bearing the K8-eYFP transgene (yellow). Embryos at E6.5 were cleared using RIM buffer, mounted with 1.42 IR silicone oil. All embryos were imaged by confocal microscopy with a ×40 water immersion objective. **(A–A‴)** whole-mount 3D reconstructions (scale bars = 50 µm); **(B–B‴)** zoomed views of the VE at the border between embryonic and extra-embryonic regions (scale bars = 20 µm). **(C–C‴)** are zooms of extra-embryonic VE (scale bars = 10 µm) and **(D–D‴)** are zooms of embryonic VE (scale bars = 10 µm). ExE, extra-embryonic; Emb, embryonic.

In addition to the difference in levels of K8 expression between embryonic and extra-embryonic VE pre and post AVE migration, 3D reconstruction of wholemount embryos highlighted a distinct subcellular localization (Figure 4). Indeed, the distribution of K8-eYFP at cell-cell junctions became discontinuous in the extra-embryonic region while the density of the network increased in the embryonic VE, both in the columnar AVE cells, where it extended to the lateral surface, and in the surrounding squamous VE cells. The dotted appearance of the K8-eYFP fluorescence in the extra-embryonic VE is reminiscent of the one observed in outer cells upon compaction (Schwarz et al., 2015). It may correspond to a maintenance of K8 only at desmosomes, while in the embryonic VE keratin filaments are retained all along cell-cell junctions. It is counter-intuitive to observe a presumably less mature keratin mesh in the least dynamic epithelium, in which cells have stable hexagonal shapes and do not intercalate (Trichas et al., 2012). It is possible that the dense actin shroud in the extra-embryonic VE hinders the recycling of keratin filaments, eventually resulting in a weakening of the network (Leube et al., 2017).

**FIGURE 5:**
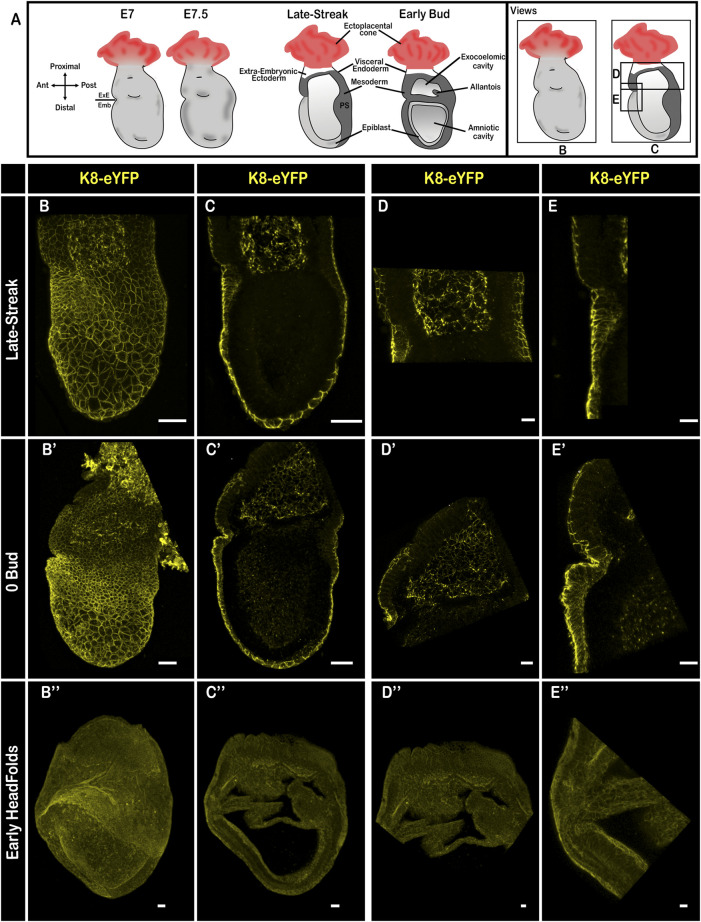
Keratin 8 organization in E7.5 mouse embryos. **(A)** Schemes of E7.5 embryo morphology, germ layers, orientation, and representation of angles of view. ExE: extra-embryonic, Emb: embryonic. **(B–E”)** Confocal images from Late-Streak **(B–E)**, 0 Bud **(B′–E′)** and Early Head Folds **(B”–E”)** fixed embryos bearing the K8-eYFP transgene (yellow). Embryos were cleared using the RIM buffer, mounted in conical wells (Ibidi) with 1.42 IR silicone oil, and imaged by confocal microscopy with a ×20 dry objective (*n* = 18). **(B,B′,B”)** whole-mount 3D reconstructions (scale bars = 50 µm); **(C,C′,C”)** 3D projections from selected Z-slices (scale bars = 50 µm); **(D,D′,D”)** 3D projection from selected Z-slices of the extra-embryonic region (scale bars = 20 µm); **(E,E,E”)** 3D projections from selected Z-slices of the anterior side at the border between embryonic and extra-embryonic regions (scale bars = 20 µm).

At E7.5 (Figure 5A), K8 marked the extra-embryonic ectoderm and mesoderm, as previously described (Saykali et al., 2019; Nahaboo et al., 2022), and was enriched at cell-cell junctions in the endoderm (Figures 5B–E,B′–E′; Supplementary Video S9), which at this stage is composed of VE only in the extra-embryonic region, and of a mixture of VE and definitive endoderm cells that intercalate between VE cells in the embryonic region (Kwon et al., 2008). Single cell RNA sequencing showed that definitive endoderm cells have a lower mean level of *Krt8* transcripts than VE at E7.25; between E7.5 and E8 *Krt8* mRNA content tends to equalize in the high range in all endoderm cells (https://marionilab.cruk.cam.ac.uk/MouseGastrulation2018/(Pijuan-Sala et al., 2019)). Live imaging at early E7.5 uncovered a salt and pepper pattern in the embryonic endoderm (Supplementary Figure S2C; Supplementary Video S6), which may correspond to transient intercalation events. At late gastrulation, when the VE is expected to have been completely displaced by definitive endoderm in the embryonic region, the level of K8 in the endoderm layer was homogenous (Figures 5B″–E″).

Prior to gastrulation, only one of the two epithelial layers of each region of the embryo has high density of keratin filaments: the VE in the embryonic region, and the ectoderm in the extra-embryonic region. One could speculate that it is sufficient to ensure robustness of the embryo wall, and that having two layers with distinct mechanical properties is favorable for the growth and shape changes that accompany gastrulation.

## Data Availability

The raw data supporting the conclusion of this article will be made available by the authors, without undue reservation.

## Supplementary material

SUPPLEMENTARY FIGURE S1 Patterns of Vimentin, F-actin, and Keratin 8 at E5.5 and E6.5 and maintenance of Keratin 8 distribution in the VE upon disruption of the actin cytoskeleton. **(A-D)** 3D reconstruction **(A,B)** and 3D projection of selected Z-slices **(C,D)** of a whole-mount E5.5 Pre-AVE migration embryo stained for F-actin (Phalloidin, grey), Keratin 8 (yellow), nuclei (DAPI, blue) and Vimentin (magenta) mounted in PBS and imaged by confocal microscopy with a ×40 water immersion objective. **(E–H)** 3D reconstruction **(E,F)** and 3D projection of selected Z-slices **(G,H)** of a whole-mount E6.5 Early-Streak embryo stained for F-actin (Phalloidin, grey), Keratin 8 (yellow), nuclei (DAPI, blue) and Vimentin (magenta) cleared with RIM buffer and mounted with 1.42 IR silicon oil to be imaged by confocal microscopy with a ×25 water immersion objective. Scale bars = 50 µm E5.5 (*n* = 4); E6.5 (*n* = 10). **(I,J)** 3D projection of selected Z-slices of a whole-mount E6.5 embryo stained for F-actin [**(I)**, Phalloidin, grey] and expressing K8-eYFP [**(J)**, yellow]; scale bar = 50 µm. White squares on the right are zooms in the extra-embryonic (top) and embryonic (bottom) regions where the fluorescence is represented as intensity levels (red indicates a high signal while blue indicates a low signal); scale bar = 10 µm. **(K)** Graph representing the average ratio (embryonic grey-value/extra-embryonic grey-value) for K8-eYFP and F-actin (*n* = 1,015 measurements from 5 embryos). Shapiro-Wilk normality test W equals 0.9581 for K8-eYFP and 0.9524 for F-actin. Unpaired *t*-test *p*-value is 0.0001. **(L)** Table of grey-values (arbitrary unit, mean ± sem) in embryonic and extra-embryonic regions, as well as their ratio for K8-eYFP and F-actin. **(M–P)** 3D reconstruction **(M,N)** and 3D projection of selected Z-slices **(O,P)** of two whole-mount K8-eYFP E6.5 embryos treated with Cytochalasin D at 5 µM for 30 min, fixed and stained for F-actin (Phalloidin, grey) and nuclei (DAPI, blue) before imaging by confocal microscopy with a ×40 water immersion objective. Scale bars = 50 µm, *n* = 8.

SUPPLEMENTARY FIGURE S2 Live imaging of K8-eYFP E5.75, E6.5, and E7.5 embryos. 3D reconstructions of stills from confocal imaging of an E5.75 K8-eYFP embryo (yellow) imaged every 15 min for a total of 120 min. **(B)** 3D reconstructions of stills from confocal imaging of an E6.5 K8-eYFP (yellow, also depicted in (Nahaboo et al., 2022) imaged every 60 min for a total of 300 min. **(C)** 3D reconstructions of stills from confocal imaging of an E7.5 K8-eYFP (yellow, also depicted in (Nahaboo et al., 2022) imaged every 60 min for a total of 180 min. Scale bars = 50 µm.

SUPPLEMENTARY VIDEO S1 3D reconstruction (first half) and Z-slices stack (second half) of a E4.25 K8-eYFP embryo stained with DAPI (nuclei) (shown in Figure 1).

SUPPLEMENTARY VIDEO S2 3D reconstructions from confocal microscopy live imaging of a E5.75 K8-eYFP (yellow) embryo. Interval time: 15 min; scale bar: 50 µm; shown in Supplementary Figure S2.

SUPPLEMENTARY VIDEO S3 3D reconstruction and Z-slices stack of an E5.5 K8-eYFP embryo at the AVE migration stage; whole embryo view with DAPI (nuclei) staining (first half) and zoom of the ExE region (second half).

SUPPLEMENTARY VIDEO S4 3D reconstruction (first half) and Z-slices stack (second half) of a post-AVE migration E5.5 K8-eYFP embryo stained with DAPI (nuclei) (shown in Figure 2).

SUPPLEMENTARY VIDEO S5 3D reconstructions from confocal microscopy live imaging of a E6.5 K8-eYFP (yellow) embryo. Interval time: 60 min; scale bar: 50 µm; shown in Supplementary Figure S2.

SUPPLEMENTARY VIDEO S6 3D reconstructions from confocal microscopy live imaging of a E7.5 K8-eYFP (yellow) embryo. Interval time: 60 min; scale bar: 50 µm, shown in Supplementary Figure S2.

SUPPLEMENTARY VIDEO S7 3D reconstruction (first half) and Z-slices stack (second half) of a Pre-Streak E6.5 K8-eYFP embryo stained with DAPI (nuclei) (shown in Figure 3).

SUPPLEMENTARY VIDEO S8 3D reconstruction (first half) and Z-slices stack (second half) of a Mid-Streak E6.5 K8-eYFP (yellow) embryo stained with DAPI (nuclei) (shown in Figure 3).

SUPPLEMENTARY VIDEO S9 3D reconstruction (first half) and Z-slices stack (second half) of a 0 Bud E7.5 K8-eYFP (yellow) embryo (shown in Figure 5).
