# Supplementary figures and images for: Regionally specific levels and patterns of keratin 8 expression in the mouse embryo visceral endoderm emerge upon anterior-posterior axis determination
Source: Front Cell Dev Biol. 2022 Dec 1;10:1037041. doi: 10.3389/fcell.2022.1037041 (PMC9751397; doi:10.3389/fcell.2022.1037041)

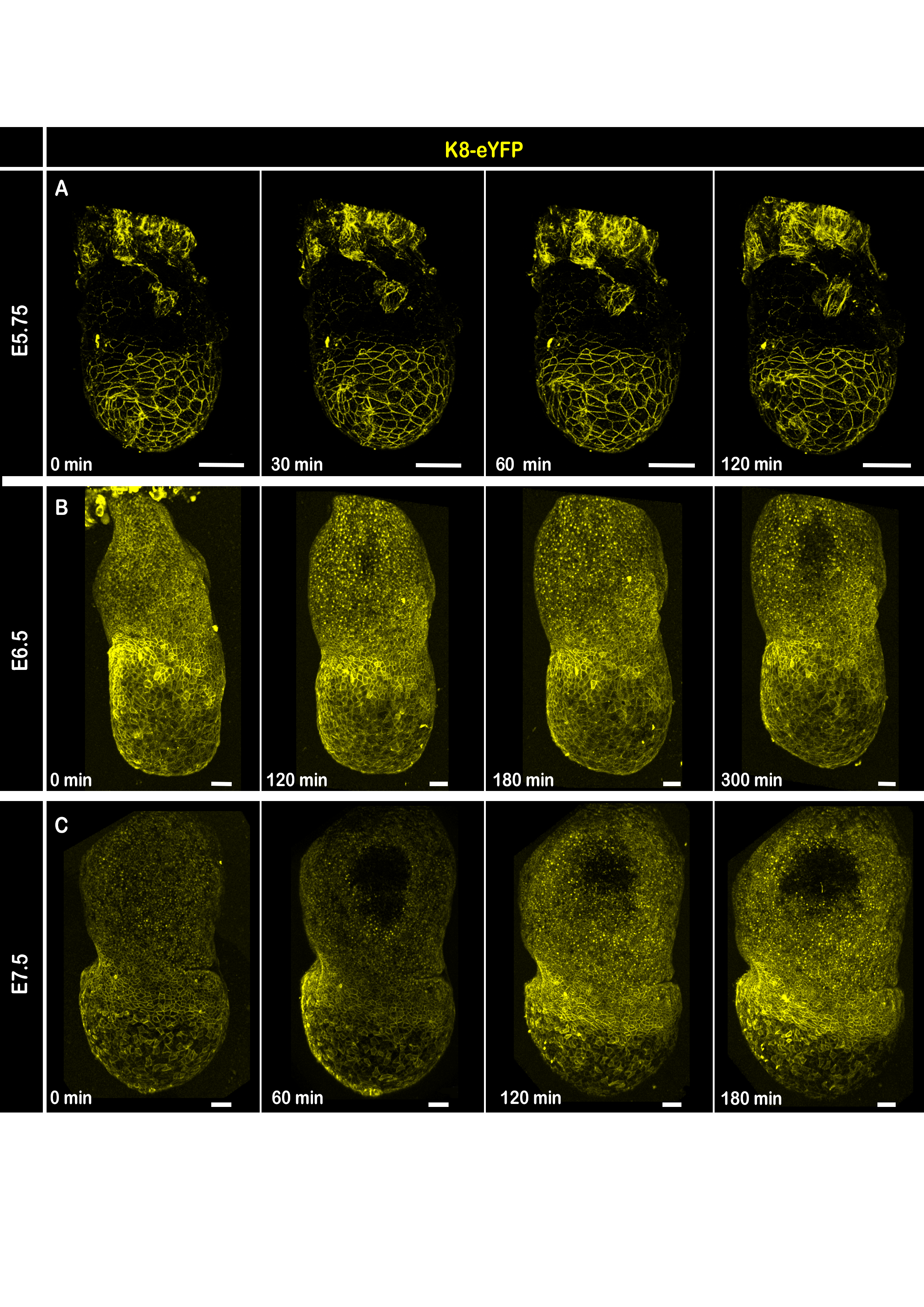

Supplement: Supplementary file 3 [file Image2.TIF]

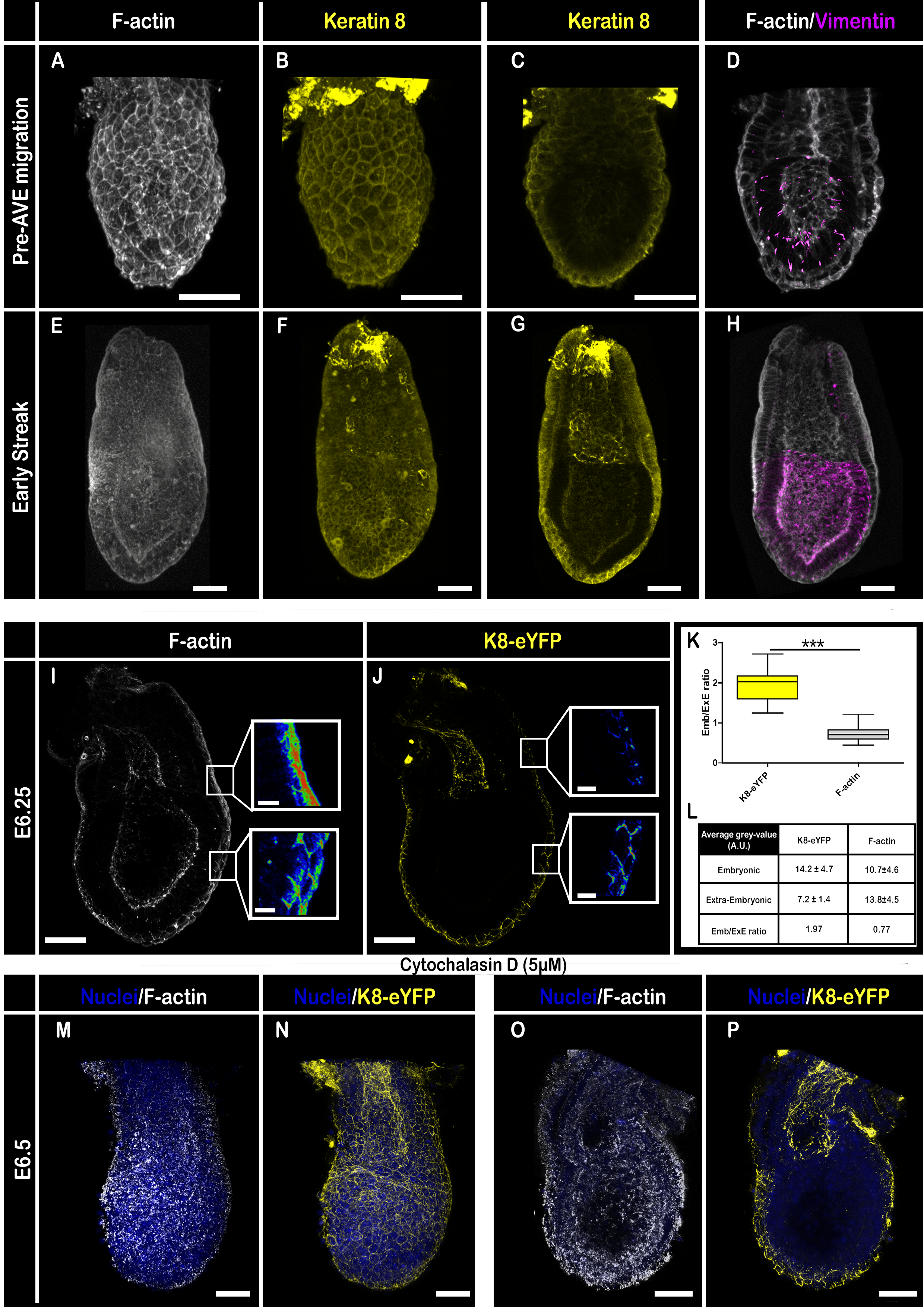

Supplement: Supplementary file 4 [file Image1.TIF]
